# Molecular Detection and Identification of Zoonotic Microsporidia Spore in Fecal Samples of Some Animals with Close-Contact to Human

**Published:** 2015

**Authors:** Zeinab ASKARI, Hamed MIRJALALI, Mehdi MOHEBALI, Zabih ZAREI, Saeideh SHOJAEI, Tahereh REZAEIAN, Mostafa REZAEIAN

**Affiliations:** 1 *Dept. of Medical Parasitology and Mycology, School of Public Health, Tehran University of Medical Sciences, Tehran, Iran*; 2 *Center for Research of Endemic Parasites of Iran (CREPI), Tehran University of Medical Sciences, Tehran, Iran*; 3 *Meshkin-Shahr Research Station, School of Public Health and National Institute of Health Research, Tehran University of Medical Sciences, Tehran, Iran*

**Keywords:** Laboratory animals, *Enterocytozoon bieneusi*, *Encephalitozoon cuniculi*, Zoonotic transmission

## Abstract

***Background:*** Microsporidia species are obligatory intracellular agents that can infect all major animal groups including mammals, birds, fishes and insects. Whereas worldwide human infection reports are increasing, the cognition of sources of infection particularly zoonotic transmission could be helpful. We aimed to detect zoonotic microsporidia spore in fecal samples from some animals with close – contact to human.

***Methods:*** Overall, 142 fecal samples were collected from animals with closed-contact to human, during 2012-2013. Trichrome – blue staining were performed and DNA was then extracted from samples, identified positive, microscopically. Nested PCR was also carried out with primers targeting SSU rRNA gene and PCR products were sequenced.

***Results:*** From 142 stool samples, microsporidia spores have been observed microscopically in 15 (10.56%) samples. *En. cuniculi* was found in the faces of 3 (15%) small white mice and 1 (10%) laboratory rabbits(totally 2.81%). Moreover, *E. bieneusi* was detected in 3 (10%) samples of sheep, 2 (5.12%) cattle, 1 (10%) rabbit, 3 (11.53%) cats and 2 (11.76%) ownership dogs (totally 7.74%). Phylogenetic analysis showed interesting data**.** This is the first study in Iran, which identified *E. bieneusi and En. Cuniculi* in fecal samples of laboratory animals with close – contact to human as well as domesticated animal and analyzed them in phylogenetic tree.

***Conclusion:*** *E. bieneusi *is the most prevalent microsporidia species in animals. Our results can also alert us about potentially zoonotic transmission of microsporidiosis.

## Introduction

Microsporidia group is an obligatory intracellular agent, which can infect all major animal groups including mammalian, birds, fishes and insects (1-3). At first, *Microsporidia* spp., were only known as causative agent of economic losses in silkworm, honeybee and fishery industries (3). With identification of microsporidiosis in human in 1959 (4) and increasing the reports of the infection in immunocompromised patients particularly in recent years, the worth of identification of infection in animals as potentially sources of zoonotic transmission, has been illuminated more than before (3). 

Although there are vast information in epidemiology and infection of microsporidia, but the manner of transmission and source of human infection have not been clearly known (5, 6). The zoonotic transmission of microsporidia has not been demonstrated yet, but this way could be concerned as a considerable reason of *Microsporidia* infection in Human populations. The species that infect human, particularly *E. bieneusi *and *Encephalitozoon *spp*., *have been reported frequently from wide range of mammalian hosts (7-10). Several studies have been carried out on laboratory, household and wild animals as well as birds, which introduced them as potentially zoonotic sources of human intestinal microsporidiosis (8, 9, 11-18). 

However, only few studies have been carried out around intestinal *Microsporidia* species in animals related to human in Iran. To date, some studies were performed on pigeon (19), cat and dog (14), but other human closed-contiguity animals have not been considered as potentially zoonotic sources. This study aimed to determine intestinal *Microsporida* species in laboratory rabbits and small white mice, ownership dogs, cats, dairy cattle and sheep. 

## Materials and Methods

***Animal stool samples***

A total of 142 fecal samples were collected from animals with closed-contact to human, containing laboratory small white mice (n=20), rabbits (n=10), ownership dogs (n=17), cats (n=26), sheep (n=30) and dairy cattle (n=39) during 2012-2013. Samples of ownership dog and cats were provided from Meshkin shahr district, Ardebil, Iran. Sheep and dairy cattle were provided from same farm in Tehran Province and finally laboratory animals sample was obtained from Animal House of School of Public Health, Tehran University of Medical Sciences. 

Samples were washed according to method that mentioned elsewhere (20). Briefly, the samples were suspended in sterile PBS (pH: 7.5) and filtrated with sterile gases for debris exclusion. Remained suspension washed three times by sterile PBS (pH: 7.5). After final centrifuging at 2500 rpm for 10 min and removing the supernatant, remained pellet of each isolate was re-suspended in sterile PBS (pH: 7.5) , formalin – PBS 5% for molecular and parasitological survey, respectively.

***Parasitology***

Slim slides of each sample, stained with trichrome - blue staining, were performed (21). All the slides were examined under light microscope with high magnifications (1000 X). Ovoid, pinkish spore that were about 1-2.5 µm were considered as *Microsporidia* spp. 

***DNA extraction***

DNA extraction was carried out for all microscopically positive and negative samples (20). Briefly, 250 µl of PBS suspended stool were transferred to 1.5 ml tube. After centrifuging in 10000 rpm for 5 min and removing the supernatant, 400 µl of lysis buffer (Tris 100mM, EDTA 10 mM, SDS 2% and 20 µg per µl of Proteinase K) and 300 µl volume of acid washed Glass beads (size 425-600 µm) were added to pellet. Every tube was shaken vigorously for 2 min and was placed in 60 °C for 4 hours. Samples were shaken every 30 min intensely. Subsequently, after centrifuging at 3000 rpm for 5 min and supernatant was collected and transferred to Bioneer stool DNA extraction kit (Bioneer Corporation, Daejeon, Korea). Purified DNA stored at -20 °C until use.

***Nested - Polymerase Chain Reaction***

Nested- PCR was carried out in final volume 25 µl, containing 2.5 µl of 10X PCR buffer, 2 mM MgCl_2_, 200µM dNTP, 1.5 unit of Taq DNA polymerase (Fermentase, Thermo Fisher Scientific, Lithuania) and 10 ρM of each primers. Amplifications were carried out in PeqLab thermocycler (PEQLAB Biotechnologie GmbH, Germany) using Primers that mentioned by Mirjalali et al.* (*20)*, *previously. Outer primers PMicF (5´- GGTTGATTCTGCCTGACG - 3´) and PMicR (5´ - CTTGCGAGC (G/A)TACTATCC - 3´) amplify 779bp fragment of *Encephalitozoon *spp*.* and *E. bieneusi *under condition: 95 °C for 5 min as initial denaturation, following 35 cycles of 94 °C for 40 sec, 55 °C for 45 sec and 72 °C for 45 sec and finally 72 °C for 5 min as final extension. Second PCR was carried out by two pairs of genus – specific internal primers including EnbF (5´- GGTAATTTGGTCTCTGTGTG - 3´) and EnbR (5´- CTACACTCCCTATCCGTTC -3´) and also EncepF (5´- AGTACGATGATTTGGTTG- 3´) and EncepR (5´- ACAACACTATATAGTCCCGTC- 3´) which amplify 440 bp and 629 bp fragments of *E. bieneusi* and *Encephalitozoon* spp., respectively. The second PCR program conditions were: initial denaturing at 95 °C for 5 min and then 25 cycles of 94 °C for 35 sec, 57 °C for 35 sec, 72 °C for 40 sec and as a final extension, 72 °C for 3 min. Sterile distillated water and a sequenced microsporida isolate which were obtained previously (20), were used as negative and positive control, respectively and run beside all the samples. Subsequently, 5µl of PCR products were electrophoresed on 1.5% agarose gel and then were visualized by ethidium bromide staining. For more confirmation of molecular results, 20 µl of each PCR product was sequenced using by ABI 3130 (California, USA) sequencer.

***Phylogenetic analysis***

Phylogenetic tree was drawn based on our isolates and some non- human isolates retrieved from GenBank database. All of isolates information and their accession numbers were summarized inTable 1.

**Table 1:** All of information about isolates, which submitted to phylogenetic tree

**Isolate**	**Host**	**Source**	**Accession Number**
	Cat	Our study	KJ414446
	Cat	Our study	KJ414447
	Cattle	Our study	KJ414443
	Dog	Our study	KJ414448
*E. bieneusi*	Sheep	Our study	KJ414444
	Rabbit	Our study	KJ414449
	Monkey	GenBank database	AF023245
	Cattle	GenBank database	AY257180
	Falcon	GenBank database	DQ793212
*En. cuniculi*	Laboratory Mouse	Our study	KJ414452
	Laboratory Mouse	Our study	KJ414445
	Laboratory Mouse	Our study	KJ414451
	Laboratory Rabbit	Our study	KJ414449
	Rabbit	GenBank database	L17072
	Canis	GenBank database	X97469
	*Mouse musculus*	GenBank database	X98467

Molecular alignment was performed using by ClustalW in Bioedit software and then phylogenetic tree was constructed using Molecular & Evolution Genetic Analysis software version 6 (MEGA 6) in Neighbor- Joining test and Tamura 3 parameter model. For calculating the reliability of the tree, Bootstrap value with 1000 replication was considered.

***Ethical approval***

All experiments on the animals, which were included in our study, were performed based on the guidelines of the Ethical Board of Tehran University of Medical Sciences, Iran, where the study was approved.

## Results

**Fig. 1:**
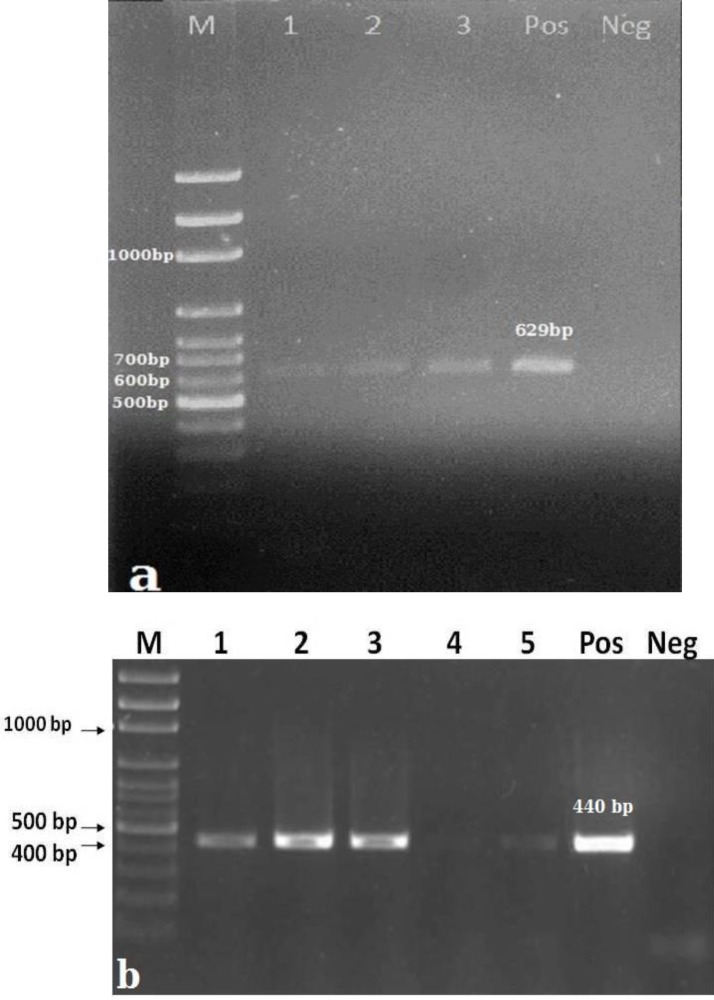
Gel electrophoresis of Nested- PCR prod ucts using by genus- specific primers; a: A 629bp fragment of *Encephalitozoon* spp., b: A 440 bp frag ment of *E. bieneusi*. Abbreviations are M: 100bp marker (Fermentase, Thermo Fisher Scientific, Lithuania), Pos: Positive control and Neg: Nega tive control

From 142 stool samples, collected from the animals, *Microsporidia* spores were observed microscopically in 15 (10.56%). Size of spores in *E. bieneusi was *1.2 – 2µm and in *En. cuniculi *was about 2-2.5 µm. All of spores were pinkish, ovoid and ellipse in form. They were also differentiable from each other in microscopic field based on spore sizes. In most of samples, parasite rate was low to moderate. Nested-PCR using new primers, which mentioned above, could confirm all of the microscopically positive samples (Fig. 1).

*E. bieneusi *and *En. cuniculi* were identified in 11 (7.74%) and 4 (2.81%) samples, respectively. *En. cuniculi *was found in the feces of 3 (15%) small white mice and 1 (10%) laboratory rabbits. Moreover, *E. bieneusi *was detected in 3 (10%) samples of sheep, 2 (5.12%) cattle, 1 (10%) rabbit, 3 (11.53%) cats and 2 (11.76%) ownership dogs. Results are summarized in Table 2.

Sequencing results and BLAST analysis confirmed the Nested-PCR results. All of samples, which were amplified with *Encephalitozoon *spp. specific primers, were characterized as *En. cuniculi.* The results of those, which were amplified with *E. bieneusi* specific primers*, *were also confirmed by sequencing. Accession numbers of submitted sequences are KJ414443 to KJ414452.

**Fig. 2:**
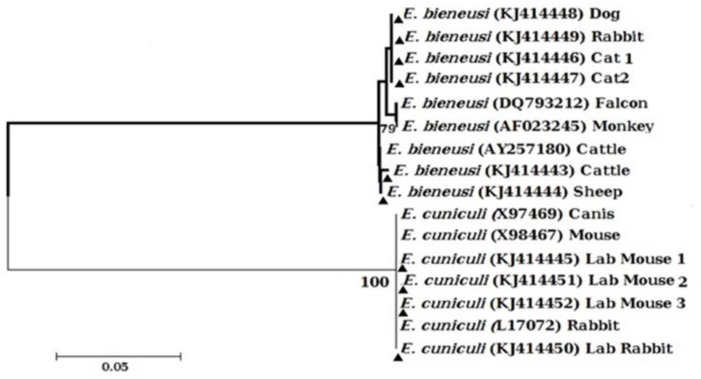
Phylogenetic tree of the SSU rRNA gene region nucleotide sequences of E. bieneusi and E. cuniculi isolates recovered from cat, ownership dog, laboratory rabbit and mouse, cattle and sheep and reference accession numbers, which retrieved from Gene Bank database. All of our isolates were marked with black filled triangle. The tree was constructed by using the Tamura3-parameter model in MEGA software version 6. The numbers above the branches indicate the percentage of bootstrap percentages. Branches without numbers have frequencies of less than 70%

According to phylogenetic tree analysis, all of isolates placed in two clades (*Encephalitozoon cuniculi *& *Enterocytozoon bieneusi). *All of* En. cuniculi, *obtained in this study, were grouped in one clade with highest Bootstrap value. *E. bieneusi* isolates which were obtained from dog, cats and rabbit were clustered together and those, which were isolated from sheep and cattle, were also placed in separated but close together (Fig. 2).

**Table 2:** Prevalence of *Microsporidia *species according to under investigation hosts and parasite species

		**Host (percent)**				
	**Cat**	**Dog**	**Sheep**	**Cattle**	**Laboratory mice**	**Laboratory rabbit**
*E. bieneusi*	11.53	11.76	10	5.12	-	10
*En. cuniculi*	-	-	-	-	15	10

## Discussion

Microsporidia infections are reported in immunocompromised patients, frequently. *E. bieneusi *and* Encephalitozoon *spp., which known as most prevalent species, could be main agents of variety range of involvements in immunocompromised individuals (22-24). Different sources have been concerned as potential agents of spreading the infection, so far (5, 25-27). In spite of the fact that zoonotic source probably play important role in human microsporidia infections, but the main transmission route of infection have not been clearly proved yet. Microsporidia spores released from stool, urine and collapsed carcasses can disperse via water and food sources. As discussed above, animals that have close – contact to human can also spread infected microsporida spores in human populations. Additionally, it is important to consider that there was no diarrhea or other characteristic sign of microsporidia infection in these animals and the parasite rate was moderate to low as well. Indeed, this subject can lead to more shedding and distributing of the spores in human society, because they were not considered as sick animals and were abandoned without receiving the treatment, subsequently. To date, study of the microsporidia infection in animals in Iran, have been carried out in two-limited study: cat, dog (14) and more recently on pigeon (19). Accessible data shows that, this study is the first assessment of intestinal microsporidiosis in vaster spectrum of domesticated and laboratory mammalian animals with close - contact to humans in Iran.

In this study, *E. bieneusi *and *En. cuniculi *spores were detected in fifteen cases. In addition, the percentage of infection among all of under assessment groups was approximately equal and prevalence of *E. bieneusi *was significantly more than *En. cuniculi.*

Our finding demonstrates that *E. bieneusi *more likely is the most prevalent microsporidia species among animals with close - contact to humans in Iran. This subject is in agreement with Pirestani and colleagues study that showed more incidence of *E. bieneusi* in pigeons (19). It is interest to mention that *E. bieneusi *in human population in Iran, particularly individuals with immune system deficiency, is also predominant species (20, 28, 29). Reports of widespread infection of *E. bieneusi* in mammalian animals as well as human reveal this fact that, even though zoonotic transmission has not been clearly proved yet, but more likely, infection via contiguity with farm, pet and laboratory animals could be one of the main routes in transmission cycle of *E. bieneusi.*

The numbers of animals infected with *En. cuniculi, *were significantly less than *E. bieneusi. *These findings can represent this fact that *Encephalitozoon *spp., has probably less effluence in domesticated animals as well as human in Iran (20, 28, 29). Additionally, all *En. cuniculi* infected cases were determined in both laboratory mice and rabbits. This finding is in agreement with other studies that emphasis on high prevalent of *En. cuniculi* in those animals (30, 31). 

Small Subunit ribosomal RNA (SSU rRNA) is one of conserved gene and can be useful in phylogenetic research of Microsporidia spp. (32, 33), According to molecular finding of SSU rRNA gene, phylogenetic analysis showed near relationship between all of *En. cuniculi *which obtained in this study. As mentioned above, all of laboratory rabbits and mice samples were collected from same laboratory, so more likely the source of all *En. cuniculi* is same. On the other hand, *E. bieneusi, which* isolated from cats, ownership dog and laboratory rabbits, showed complete proximity in this fragment of SSU rRNA gene. Although sampling place of cat and ownership dog was equal and probably they were infected from same source, but the interesting point is similarity of laboratory rabbit isolates with those, which were isolated from cat and ownership dog. Furthermore, it is interest to mention that although cattle and sheep isolates have trivial differences with each other in sequence analysis at the positions but they were grouped more near together than those isolated from cats, laboratory rabbit and dog and this fact could be related to place of sampling. As mentioned former, cattle and sheep isolates, obtained from same dairy farm and different from other isolates, geographically. 

## Conclusion

*E. bieneusi *probably is the most prevalent microsporidia species in animal hosts as well as human in Iran and more likely infected animals with close- contact to human play an irrefutable role in transmission cycle of Microsporidia spp., particularly *E. bieneusi*. Our results also show that animals especially domesticated ones, without clinical sign could be importance in zoonotic transmission of microsporidia species. 

## References

[1] Sprague V, Becnel JJ, Hazard EI (1992). Taxonomy of phylum microspora. Crit Rev Microbiol.

[2] Canning EU, Lom J, Dykova I (1986). The microsporidia of vertebrates. JBM.

[3] Wittner M, Wittner M, Weiss L (1999). Historic perspective on the microsporidia: expanding horizons. The Microsporidia and Microsporidiosis.

[4] Matsubayashi H, Koike I, Mikata I, Takei H, Hagiwara S (1959). A case of Encephalitozoon-like infection in man. Arch Pathol.

[5] Didier ES, Stovall ME, Green LC (2004). Epidemiology of microsporidiosis: sources and modes of transmission. Vet Parasitol.

[6] Mathis A, Weber R, Deplazes P (2005). Zoonotic potential of the microsporidia. Clin Microbiol Rev.

[7] Mathis A, Breitenmoser AC, Deplazes P (1999). Detection of new Enterocytozoon genotypes in faecal samples of farm dogs and a cat. Parasite.

[8] Haro M, Izquierdo F, Henriques-Gil N (2005). First detection and genotyping of human-associated microsporidia in pigeons from urban parks. Appl Environ Microbiol.

[9] Fayer R, Santin MJ, Trout M (2003). First detection of microsporidia in dairy calves in North America. Parasitol Res.

[10] Sak B, Kvac M, Hanzlikova D, Cama V (2008). First report of Enterocytozoon bieneusi infection on a pig farm in the Czech Republic. Vet Parasitol.

[11] Lores B, Del Aguila C, Arias C (2002). Enterocytozoon bieneusi (microsporidia) in faecal samples from domestic animals from Galicia, Spain. Mem Inst Oswaldo Cruz.

[12] Deplazes P, Mathis A, Baumgartner R, Tanner I, Weber R (1996). Immunologic and molecular characteristics of Encephalitozoon-like mic-rosporidia isolated from humans and rabbits indicate that Encephalitozoon cuniculi is a zoonotic parasite. Clin Infect Dis.

[13] Thomas C, Finn M, Twigg L, Deplazes P, Thompson RC (1997). Microsporidia (Enceph-alitozoon cuniculi) in wild rabbits in Australia. Aust Vet J.

[14] Jamshidi S, Tabrizi AS, Bahrami M, Momtaz H (2012). Microsporidia in household dogs and cats in Iran; a zoonotic concern. Vet Parasitol.

[15] Slodkowicz-Kowalska A (2009). Animal reservoirs of human virulent microsporidian species. Wiad Parazytol.

[16] Black SS, Steinohrt LA, Bertucci DC, Rogers LB, Didier ES (1997). Encephalitozoon hellem in budgerigars (Melopsittacus undulatus). Vet Pathol.

[17] Sak B, Kasickova D, Kvac M, Kvetonova D, Ditrich O (2010). Microsporidia in exotic birds: intermittent spore excretion of Encephalitozoon spp. in naturally infected budgerigars (Melopsittacus undulatus). Vet Parasitol.

[18] Abe N, Kimata I (2010). Molecular survey of Enterocytozoon bieneusi in a Japanese porcine population. Vector Borne Zoonotic Dis.

[19] Pirestani M, Sadraei J, Forouzandeh M (2013). Molecular characterization and genotyping of human related microsporidia in free-ranging and captive pigeons of Tehran, Iran. Infection, Genetics and Evolution.

[20] Mirjalali H, Mohebali M, Mirhendi H (2014). Emerging intestinal microsporidial infection in HIV+/AIDS patients in Iran: microscopical and molecular detection. Iran J Parasitol.

[21] Ryan NJ, Sutherland G, Coughlan K (1993). A new trichrome-blue stain for detection of microsporidial species in urine, stool, and nasopharyngeal specimens. J Clin Microbiol.

[22] Didier ES (2005). Microsporidiosis: an emerging and opportunistic infection in humans and animals. Acta Trop.

[23] Dascomb K, Frazer T, Clark RA, Kissinger P, Didier E (2000). Microsporidiosis and HIV. J Acquir Immune Defic Syndr.

[24] Franzen C, Muller A (2001). Microsporidiosis: human diseases and diagnosis. Microbes Infect.

[25] Hoffman RM, Wolk DM, Spencer SK, Borchardt MA (2007). Development of a method for the detection of waterborne microsporidia. J Microbiol Methods.

[26] Decraene V, Lebbad M, Botero-Kleiven S, Gustavsson AM, Lofdahl M (2011). First reported foodborne outbreak associated with microspo-ridia, Sweden, October 2009. Epidemiol Infect.

[27] Deplazes P, Mathis A, Weber R (2000). Epidemiology and zoonotic aspects of microsporidia of mammals and birds. Contrib Microbiol.

[28] Agholi M, Hatam GR, Motazedian MH (2013). Microsporidia and coccidia as causes of persistence diarrhea among liver transplant children: incidence rate and species/genotypes. Pediatr Infect Dis J.

[29] Agholi M, Hatam GR, Motazedian MH (2013). HIV/AIDS-associated opportunistic protozoal diarrhea. AIDS Res Hum Retroviruses.

[30] Wasson K, Peper RL (2000). Mammalian microspo-ridiosis. Vet Pathol.

[31] Sak B, Ditrich O (2005). Humoral intestinal immunity against Encephalitozoon cuniculi (Microsporidia) infection in mice. Folia Parasitol (Praha).

[32] Tay WT, O'Mahony EM, Paxton RJ (2005). Complete rRNA gene sequences reveal that the microsporidium Nosema bombi infects diverse bumblebee (Bombus spp.) hosts and contains multiple polymorphic sites. J Eukaryot Microbiol.

[33] Dong SN, Shen ZY, Xu L, Zhu F (2010). Sequence and phylogenetic analysis of SSU rRNA gene of five microsporidia. Curr Microbiol.

